# Modification of gingival proteoglycans by reactive oxygen species: potential mechanism of proteoglycan degradation during periodontal diseases

**DOI:** 10.1080/10715762.2021.2003351

**Published:** 2021-11-25

**Authors:** Ryan Moseley, Rachel J. Waddington

**Affiliations:** Regenerative Biology Group, Oral and Biomedical Sciences, School of Dentistry, Cardiff Institute of Tissue Engineering and Repair (CITER), College of Biomedical and Life Sciences, Cardiff University, Cardiff, UK

**Keywords:** Gingiva, proteoglycans, glycosaminoglycans, extracellular matrix, reactive oxygen species, periodontal diseases

## Abstract

Reactive oxygen species (ROS) overproduction and oxidative stress are increasingly being implicated in the extracellular matrix (ECM) degradation associated with chronic inflammatory conditions, such as periodontal diseases. The present study investigated the effects of ROS exposure on the proteoglycans of gingival tissues, utilizing an *in vitro* model system comprised of supra-physiological oxidant concentrations, to ascertain whether gingival proteoglycan modification and degradation by ROS contributed to the underlying mechanisms of ECM destruction during active gingivitis. Proteoglycans were purified from ovine gingival tissues and exposed to increasing H_2_O_2_ concentrations or a hydroxyl radical (·OH) flux for 1 h or 24 h, and ROS effects on proteoglycan core proteins and sulfated glycosaminoglycan (GAG) chains were assessed. ROS were capable of degrading gingival proteoglycans, with ·OH species inducing greater degradative effects than H_2_O_2_ alone. Degradative effects were particularly manifested as amino acid modification, core protein cleavage, and GAG chain depolymerization. Proteoglycan core proteins were more susceptible to degradation than GAG chains with H_2_O_2_ alone, although core proteins and GAG chains were both extensively degraded by ·OH species. Proteoglycan exposure to ·OH species for 24 h induced significant core protein amino acid modification, with decreases in glutamate, proline, isoleucine, and leucine; and concomitant increases in serine, glycine, and alanine residues. As clinical reports have previously highlighted proteoglycan core protein degradation during chronic gingivitis, whereas their sulfated GAG chains remain relatively intact, these findings potentially provide further evidence to implicate ROS in the pathogenesis of active gingivitis, complementing the enzymic mechanisms of periodontal tissue destruction already established.

## Introduction

Periodontal diseases, comprising gingivitis and periodontitis, typically describe a range of inflammatory conditions which affect both the soft and hard tissues of the periodontium. They are regarded as the most common disease of mankind, leading to huge economic burdens for healthcare providers [1]. As its prevalence is also associated with risk factors such as age and diabetes, projections estimate further escalations in the incidence of periodontal diseases with ever-increasing age demographics and diabetic rates Worldwide. Dental plaque accumulation and the uncontrolled formation of bacterial biofilms predominantly composed of pathogenic Gram-negative bacterial species, such as *Porphyromonas gingivalis*, are commonly associated with the initiation and progression of periodontal diseases [2]. However, subsequent perturbations and the sustained dysregulated nature of the host immuno-inflammatory response are believed to be primarily responsible for the tissue damage associated with periodontal tissues, following stimulation by bacterial-derived mediators [3–8]. Consequently, in addition to proteases and other enzymes originating from microbial and host cell sources [3,7,9–11], excessive reactive oxygen species (ROS) production by resident inflammatory cells and the resultant oxidative stress, are increasingly implicated as supplementary mechanisms responsible for host connective tissue destruction during periodontal diseases [12–16].

Oxidative stress refers to the balance between ROS production and cellular/tissue antioxidant defense mechanisms. ROS are generated via a wide range of cellular mechanisms, although infiltrating neutrophils and macrophages are well-recognized as the principal source of superoxide radical (O_2_·^−^), hydrogen peroxide (H_2_O_2_), and the hydroxyl radical (·OH) species during periodontal diseases in response to periodontal pathogens and their products, or as a consequence of genetic disorders which alter neutrophil respiratory burst functions [17–21]. Although ROS overproduction effectively enhances the bactericidal activity of inflammatory cells against periodontal pathogens; and ROS levels within tissues are tightly regulated by enzymic and non-enzymic antioxidant defense mechanisms, excessive ROS production during active periodontal disease can overwhelm endogenous antioxidant capabilities, causing indiscriminate biomolecular damage to DNA, proteins, and lipids; leading to altered cellular functions [12,22–26]. Furthermore, ROS are also capable of directly and indirectly (via proteolytic activation) degrading extracellular matrix (ECM) components, including collagen, proteoglycans, and hyaluronan [12,27]. Therefore, chronic inflammation, excessive ROS production, and oxidative stress are emerging as key contributors to the host connective tissue damage associated with periodontal disease pathology.

Proteoglycans represent a large family of macromolecules, consisting of at least one poly-anionic glycosaminoglycan (GAG) chain attached to a core protein. Numerous functions have now been attributed to the various proteoglycan species, including interaction with other ECM components, the sequestration of growth factors, the regulation of collagen fibrillogenesis; and in orchestrating cell attachment, migration, and proliferation responses, with these functions mediated via the core proteins or GAG chains [28]. The proteoglycan species within periodontal tissues have been isolated and characterized from numerous sources [29], with the small leucine-rich (SLRP), dermatan sulfate-substituted proteoglycans, decorin, and to a lesser extent, biglycan; being identified as components of the gingival ECM [30–38]. Furthermore, other SLRPs, such as keratan sulfate-containing proteoglycans, lumican, and fibromodulin, have since been localized within gingival tissues [37,38]. Another integral proteoglycan in gingiva is the large aggregating, chondroitin sulfate/dermatan sulfate proteoglycan, versican [34]. The proteoglycans present in the gingival epithelium have further been characterized as predominantly consisting of heparan sulfate-containing syndecans; in addition to the SLRPs, decorin, biglycan, fibromodulin, and lumican, in lesser quantities [30–32,35,37,39–41].

Therefore, it is apparent that the degradation of gingival proteoglycans during periodontal diseases would lead to a significant loss in structural integrity and altered functions in these tissues [12,27]. Our previous work into the mechanisms by which ROS (O_2_·^−^, H_2_O_2_ and ·OH) modify and degrade periodontal proteoglycans and GAGs, has demonstrated that the sulfated GAGs commonly found in periodontal tissues, such as chondroitin 4-sulfate, dermatan sulfate, and heparan sulfate, are more resistant to the modification of hexuronic acid and hexosamine residues and subsequent GAG chain depolymerization by ROS, compared to the high molecular weight, non-sulfated GAG, hyaluronan [42–44]. Furthermore, studies have shown that these ROS are also capable of degrading the chondroitin sulfate-rich SLRPs (decorin and biglycan) present within the alveolar bone *in vitro*, with proteoglycan degradation being particularly manifested as amino acid modification and core protein cleavage, with the GAG chains being less susceptible to ROS modification and degradation [45]. In contrast, few studies have focused on the effects of ROS on the proteoglycan species within gingival tissues, although a reduction in the specific viscosity and molecular size of porcine gingival proteoglycans has been reported, following ·OH exposure *in vitro* [46]. Therefore, utilizing an *in vitro* model system composed of supra-physiological (mM) oxidant concentrations beyond those expected *in vivo* [45,47,48], this study aimed to provide a more detailed investigation of the mechanisms by which ROS, specifically H_2_O_2_ and ·OH, modify and degrade the core proteins and GAG chains comprising gingival proteoglycans; to ascertain whether the ROS-induced degradation of gingival proteoglycans contributes to the underlying mechanisms of ECM destruction associated with active gingivitis and periodontal diseases overall. Ovine gingiva was selected as a proteoglycan source, due to its availability and since periodontal disease in sheep (“broken mouth” disease) has previously provided a useful model for the study of periodontal disease pathology in humans [49,50].

## Materials and methods

### Isolation and purification of gingival proteoglycans

Proteoglycans were isolated from ovine gingivae, dissected from the heads of 9-month-old Welsh Mule breed sheep (*n = 6*), with relatively healthy dentition, obtained from a local abattoir. Gingival proteoglycans were isolated and purified by a modified method to that previously described for the isolation of proteoglycans from alveolar bone [45]. Briefly, the dissected gingival tissue was pooled from all animals and washed in phosphate-buffered saline (PBS), to remove the blood and other oral debris. The pooled gingival tissues were successively washed twice in ethanol, followed by diethyl ether (both ThermoFisher Scientific, UK); and left at room temperature for residual solvent evaporation. The ECM from the pooled gingivae was extracted into 4 M guanidinium chloride, 0.05 M sodium acetate buffer, pH 5.9, containing protease inhibitors, benzamidine hydrochloride (5 mM), iodoacetic acid (1 mM), and N-ethylmaleimide (5 mM) (all Sigma, UK), at 4 °C for 48 h, under constant agitation (30 mL buffer/g of gingiva).

The lyophilized ECM extract from the pooled gingivae was dissolved to a concentration of 10 mg/mL in 7 M urea (ThermoFisher Scientific), 0.05 M sodium acetate buffer, pH 6.8. Following centrifugation (1800 *g*, 10 min) to remove any insoluble material, solubilized extracts were applied to a Q-Sepharose (HiLoad 16/10) anion exchange column incorporated into a Fast Performance Liquid Chromatography (FPLC) System (GE Healthcare, UK) [45]; and a 0–2 M linear sodium chloride (ThermoFisher Scientific) concentration gradient applied to elute the proteoglycan-rich fractions (4 mL). Elution profiles were monitored at 280 nm and the pooled proteoglycan-rich fraction was identified by cellulose acetate electrophoresis, as described below. Proteoglycan-rich fractions were dialyzed and recovered by lyophilization, as described above; and further separated by Resource Q anion exchange column (GE Healthcare), incorporated into the FPLC System; and a 0–2 M linear sodium chloride concentration gradient was applied to elute the proteoglycan-rich fractions (1 mL). Elution profiles were again monitored at 280 nm and the pooled proteoglycan-rich fractions were confirmed by cellulose acetate electrophoresis, as described below. The proteoglycan-rich fractions were dialyzed and recovered by lyophilization, as described above.

### Degradation of gingival proteoglycans by reactive oxygen species

To examine the effects induced by exposing gingival proteoglycans to ROS, the purified proteoglycans were exposed to increasing concentrations of H_2_O_2_ or to a ·OH flux, generated by the reaction of H_2_O_2_ and Fe^3+^ [45,51]. Proteoglycans were subjected to increasing concentrations of H_2_O_2_ for a prolonged period of 24 h. However, due to the highly reactive nature of ·OH species, gingival proteoglycans were subjected to a ·OH flux for 1 h and 24 h. Short and prolonged periods of exposure (1 h and 24 h, respectively) were studied, in order to reflect the possible physiological conditions occurring during periodontal diseases.

Reaction mixtures (total volume, 200 µL), were established containing gingival proteoglycans at a final concentration of 0.5 mg/mL in 25 mM sodium acetate buffer, pH 5.6, containing 80 mM sodium chloride. To each reaction mixture, H_2_O_2_ (ThermoFisher Scientific), was added at a final concentration of 20 mM or 60 mM. An ·OH flux was generated by the addition of H_2_O_2_ (60 mM) and FeCl_3_ (1.67 mM, Sigma). Reaction mixtures containing gingival proteoglycans (0.5 mg/mL) only; and gingival proteoglycans (0.5 mg/mL) with H_2_O_2_ (60 mM), FeCl_3_ (1.67 mM); and the established ·OH scavenger and transition metal ion chelator, thiourea (33 mM, Sigma), served as controls. Reaction mixtures containing H_2_O_2_ alone were incubated at 37 °C for 24 h only, whilst experimental and control reaction mixtures involving ·OH flux generation were incubated at 37 °C for 1 h and 24 h. For 1 h incubations, generated ·OH fluxes were terminated by the addition of the Fe^3+^ chelator, desferrioxamine mesylate (6.67 mM, Sigma), in 25 mM sodium acetate buffer, pH 5.6, containing 80 mM sodium chloride. Following incubation, reaction mixtures were lyophilized and dissolved in double-distilled water to a final proteoglycan concentration of 15 mg/mL. Reaction mixtures were stored at −20 °C until required for analysis.

### Gel filtration chromatography

Gel filtration chromatography was performed to assess the fragmentation of the gingival proteoglycans to lower molecular weight products when subjected to ROS over 1 h and 24 h. Aliquots (25 µL) of each reaction mixture were applied to a Superdex 75HR 10/30 column (fractionation range 3–70 kDa), incorporated into the FPLC System; and eluted with 2 M guanidinium chloride, 0.5 M sodium acetate buffer, pH 6.8, containing 0.35 M sodium chloride [45]. Fractions (1 mL) were collected and assayed for hexuronic acid content, as previously described [42–45,52]. The assessment of gingival proteoglycan degradation by gel filtration chromatography was performed on *n* = 3 independent occasions for each experimental group.

### Cellulose acetate electrophoresis

Cellulose acetate electrophoresis was performed in order to qualitatively and quantitatively determine the GAG constituents of the gingival proteoglycans and the effects of ROS exposure upon GAG content. Aliquots (2 µL) of each reaction mixture were applied to cellulose diacetate sheets (78 × 150 mm, Electrophor, Shandon Southern, UK) in 0.2 M calcium acetate buffer (Sigma), pH 7.2; and separated at 0.6 mA per cm width of the sheet for 4.5 h [45,53]. A commercially available GAG standard mixture (Sigma), consisting of hyaluronan (HA), heparan sulfate (HS), dermatan sulfate (DS), chondroitin 4-sulfate (C4S), and chondroitin 6-sulfate (C6S), each at 50 µg/mL concentrations, were also included for all cellulose acetate electrophoretic separations [45,53]. Separated bands were stained with 0.05% Alcian blue (Sigma), containing 3% acetic acid and 50 mM magnesium chloride (Sigma), pH 3.9, with excess stain removed with 1% acetic acid, containing 50 mM magnesium chloride, pH 3.9. Following separation, GAG constituents were identified according to their electrophoretic mobility versus the respective components of the GAG standard mixture [53]. The GAG contents of each reaction mixture consisting of intact gingival proteoglycans or those exposed to ROS were calculated by densitometry, using a Chromoscan 3 Densitometer at 626 nm (Joyce-Loebl, UK). Densitometric scans were performed in triplicate for each run and the % loss in GAG contents was calculated, as determined from the integral peak area of each sample analyzed versus untreated gingival proteoglycan controls [45]. The quantification of % GAG loss following ROS exposure was performed on *n* = 3 independent occasions.

### Amino acid analysis

Amino acid analysis was performed to qualitatively and quantitatively determine the amino acid constituents of the gingival proteoglycan core proteins and to identify those amino acids susceptible to modification by ROS. Following the incubation of experimental and control reaction mixtures at 37 °C for 24 h, aliquots (100 µL) of each reaction mixture were desalted using PD-10 columns (GE Healthcare), using double-distilled water as an eluent and lyophilized. Samples were hydrolyzed in 6 M HCl (1 mL, ThermoFisher Scientific), under vacuum at 108 °C for 18 h. The separation and characterization of the individual amino acids were performed on a Pickering cation exchange amino acid analysis column, integrated into a High Performance Liquid Chromatography (HPLC) System (Dionex, UK). Amino acids were detected by the reaction of post-column eluent with ninhydrin at 55 °C and the absorbance was measured at 540 nm. Amino acid concentrations were calculated by comparison with the absorbance values obtained from a standard amino acid mixture for protein hydrolysates (Sigma) [45]. Amino acid analysis of gingival core protein compositions was performed on one occasion, with results expressed as residues per 1000.

### Statistical analysis

The quantification of % GAG loss following ROS exposure was performed on *n* = 3 independent occasions, in triplicate. Statistical analyses were performed using GraphPad (GraphPad Software, CA, USA). Data were expressed as mean ± standard deviation (SD) and statistically compared using Analysis of Variance (ANOVA), with post-hoc Tukey test. Statistical significance was considered at *p* < 0.05.

## Results

### Isolation and purification of proteoglycans from ovine gingival tissues

The isolation and purification procedures described yielded proteoglycan extracts associated with the ECM of pooled gingival tissues. Typical 280 nm elution profiles for the sequential separation and purification of proteoglycans from gingival tissues by Q-Sepharose (HiLoad 16/10) and Resource Q anion exchange chromatography are shown in (Figure S1(A) and S2(A)), respectively. The elution profiles obtained were typical of those previously reported for the purification of proteoglycans from other oral tissues, such as alveolar bone, dentin, and dental pulp [45,53]. From the Q-Sepharose (HiLoad 16/10) profiles obtained, four major fractions (I–IV) were obtained (Supplementary Figure 1(A)), with fraction III being identified as the proteoglycan-rich fraction for the isolated gingival extracts, as determined following cellulose acetate electrophoretic separation and Alcian blue staining (Supplementary Figure 1(B)). Four major fractions (I–IV) were also resolved following the further purification of the partially purified gingival proteoglycan extracts by Resource Q anion exchange chromatography (Supplementary Figure 2(A)), with fraction III again being recognized as the proteoglycan-rich fraction, following cellulose acetate electrophoretic separation and Alcian blue staining (Supplementary Figure 2(B)).

### ROS effects on gingival proteoglycan molecular size by gel filtration chromatography

**Figure 1.**
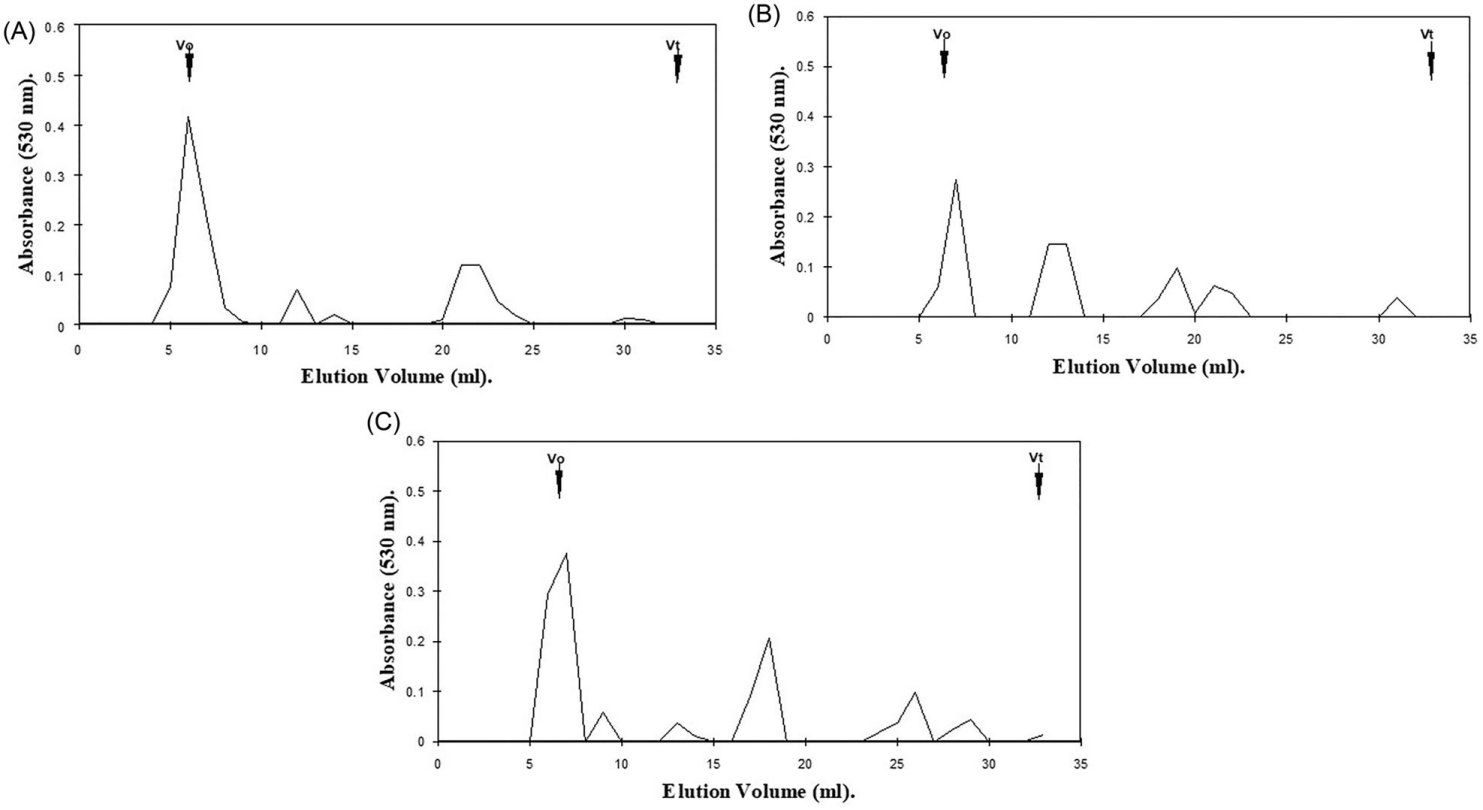
Hexuronic acid profile assessment for gingival proteoglycan molecular size, following ROS treatment for 1 h and separation by Superdex 75HR 10/30 gel filtration chromatography, incorporated into a FPLC System. (A) Untreated gingival proteoglycan controls. (B) Gingival proteoglycans exposed to an ·OH flux (60 mM H_2_O_2_, 1.67 mM FeCl_3_). (C) Gingival proteoglycans exposed to an ·OH flux (60 mM H_2_O_2_, 1.67 mM FeCl_3_), with ·OH scavenger and transition metal ion chelator, thiourea (33 mM). *V_o_*, column void volume; *V_t_*, total column volume. *n* = 3 independent experiments. For all analyses, chromatographic profiles from one representative experiment of three are shown.

**Figure 2.**
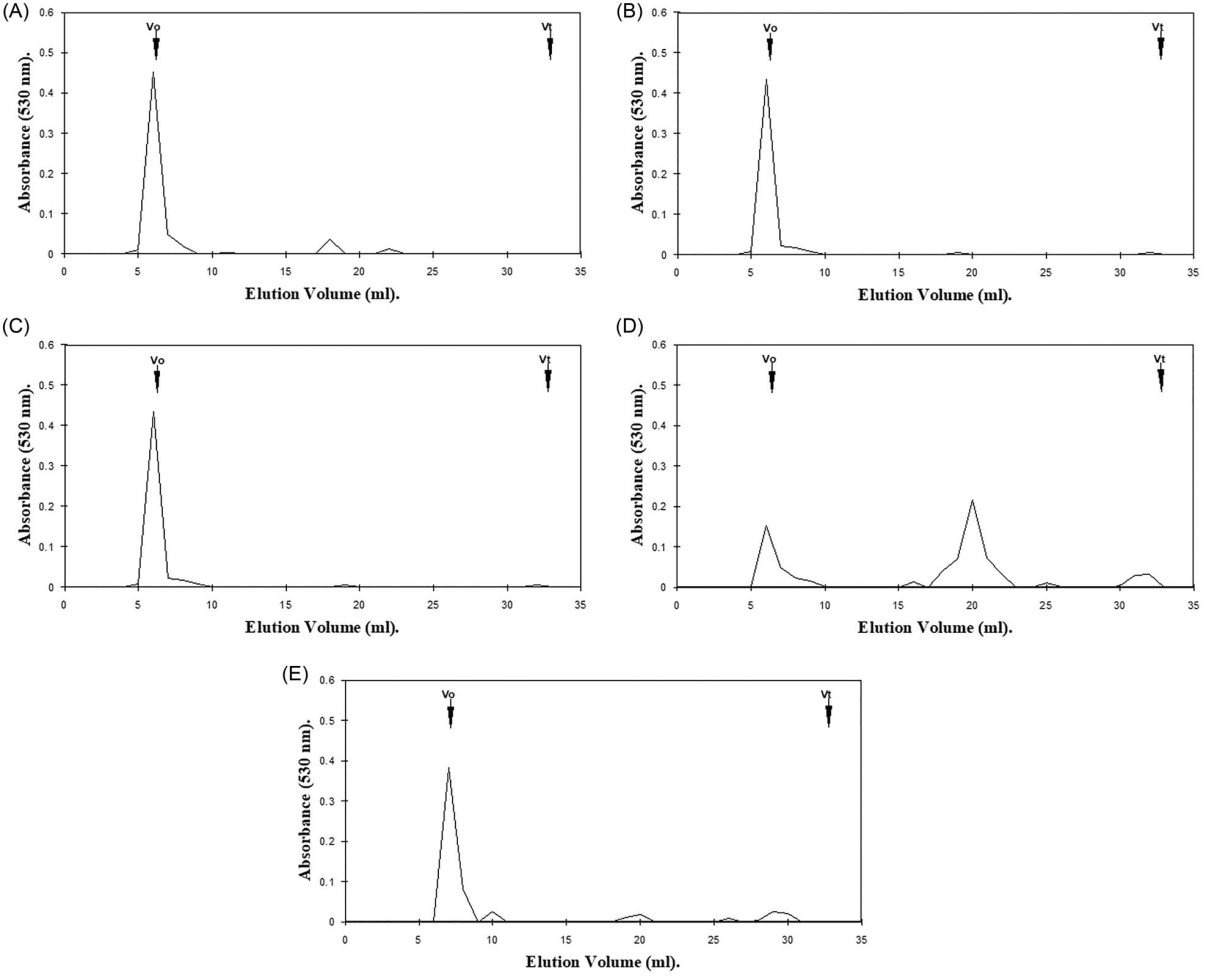
Hexuronic acid profile assessment for gingival proteoglycan molecular size, following ROS treatment for 24 h and separation by Superdex 75HR 10/30 gel filtration chromatography, incorporated into a FPLC System. (A) Untreated gingival proteoglycan controls. (B, C) Gingival proteoglycans exposed to 20 mM and 60 mM H_2_O_2_, respectively. (D) Gingival proteoglycans exposed to an ·OH flux (60 mM H_2_O_2_, 1.67 mM FeCl_3_). (E) Gingival proteoglycans exposed to an ·OH flux (60 mM H_2_O_2_, 1.67 mM FeCl_3_), with ·OH scavenger and transition metal ion chelator, thiourea (33 mM). *V_o_*, column void volume; *V_t_*, total column volume. *n* = 3 independent experiments. For all analyses, chromatographic profiles from one representative experiment of three are shown.

The hexuronic acid profiles obtained following gel filtration chromatography of gingival proteoglycans following ROS treatment for 1 h and 24 h are presented in (Figures 1 and 2), respectively. The elution profile for gingival proteoglycans exposed to an ·OH flux for 1 h demonstrated a certain degree of depolymerization, as determined by the large reduction in the hexuronic acid content of the proteoglycan parent peak near the void volume (*V_o_*, Figure 1(B)), compared to the elution profile of the untreated proteoglycan control (Figure 1(A)). Increased detection of hexuronic acid material was also identified eluting at lower molecular weights toward the total volume (*V_t_*) of the column, for the gingival proteoglycans exposed to the ·OH flux (Figure 1(B)). The inclusion of ·OH scavenger and transition metal ion chelator, thiourea, partly restored the parent peak height of the gingival proteoglycans at *V_o_* to that detected for the untreated proteoglycans (Figure 1(C)), implying that thiourea had reduced proteoglycan degradation by the ·OH flux.

Gingival proteoglycans subjected to increasing concentrations of H_2_O_2_ over 24 h exhibited little degradation to lower molecular weight products (Figure 2(B,C)), in comparison with the parent peak heights of the untreated gingival proteoglycan control, which remained relatively unchanged (Figure 2(A)). However, in the presence of an ·OH flux for 24 h, significant reductions in the hexuronic acid content of the parent peak near *V_o_* were evident, with corresponding increases in the detection of hexuronic acid material eluting toward *V_t_* (Figure 2(D)). It was further noted that the extent of gingival proteoglycan degradation by the ·OH flux over 24 h was much greater than that observed following 1 h ·OH exposure (Figure 1(B)). Thiourea again reestablished the parent peak height of the gingival proteoglycans near *V_o_* to an extent and also reduced the levels of lower molecular weight hexuronic acid products detected (Figure 2(E)).

### ROS effects on gingival proteoglycan GAG chains by cellulose acetate electrophoresis

**Figure 3.**
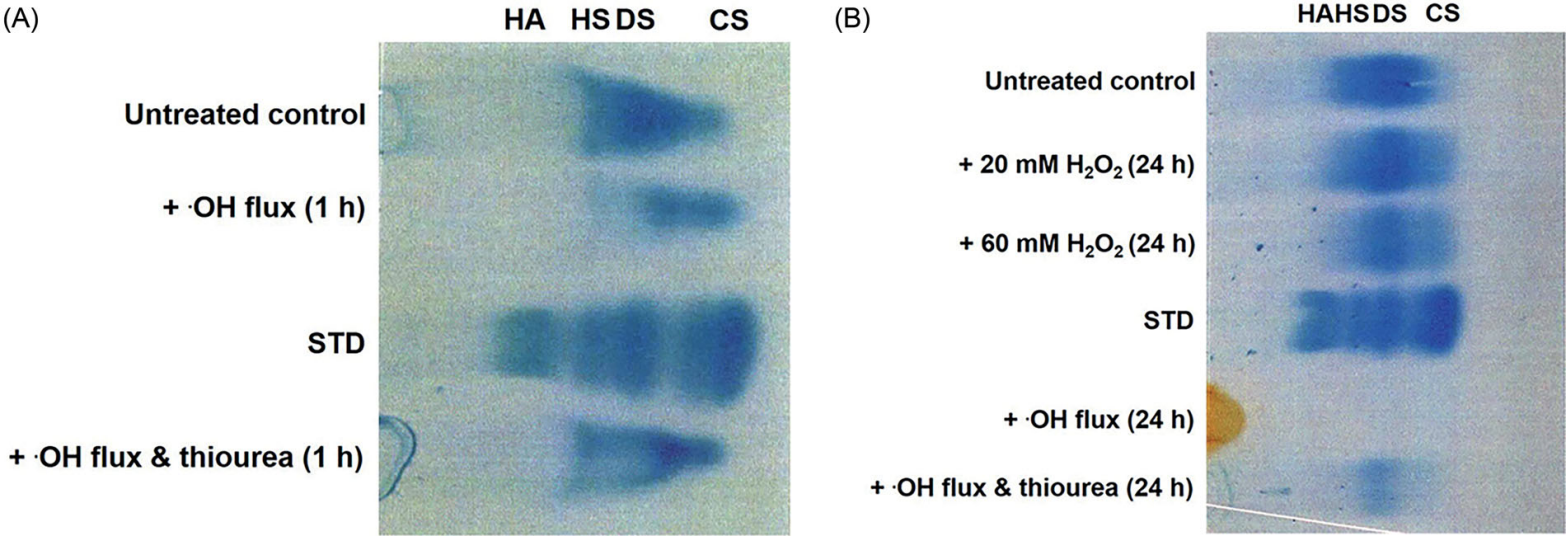
Cellulose acetate electrophoresis assessment of GAG content in gingival proteoglycans following ROS treatment for (A) 1 h and (B) 24 h. STD, commercial GAG standard; HA, hyaluronan; HS, heparan sulfate; DS, dermatan sulfate; CS, chondroitin sulfate. *n* = 3 independent experiments. For all analyses, electrophoretic profiles from one representative experiment of three are shown.

The cellulose acetate electrophoresis analysis of gingival proteoglycans following ROS exposure for 1 h and 24 h, are shown (Figure 3(A,B)), respectively. The predominant GAG constituent of untreated ovine gingival proteoglycans was identified as dermatan sulfate, with lesser quantities of chondroitin sulfate and heparan sulfate. Gingival proteoglycan exposure to an ·OH flux for 1 h resulted in a significant reduction in GAG content (∼37%, *p* < 0.001), compared to untreated gingival proteoglycans (Figure 3(A)) and Table 1. However, supplementation with thiourea significantly attenuated the percentage loss in GAG content by gingival proteoglycans subjected to the ·OH flux (∼10%, *p* < 0.001, Figure 3(A)) and Table 1. However, GAG loss in the presence of thiourea was still found to be significantly higher than untreated gingival proteoglycans alone (*p* < 0.001, Figure 3(A)) and Table 1.

**Table 1.** Total % loss in GAG content by gingival proteoglycans exposed to ROS for 1 h and 24 h. Results are presented as mean ± SD, *n* = 3 independent experiments.

ROS Treatment	% Loss of GAG Content
+ ·OH flux (1 h)	36.77 ± 0.33*
+ ·OH flux and thiourea (1 h)	9.94 ± 0.21*
+ 20 mM H_2_O_2_ (24 h)	0.18 ± 0.54
+ 60 mM H_2_O_2_ (24 h)	13.25 ± 0.36*
+ ·OH flux (24 h)	82.55 ± 0.80*
+ ·OH flux and thiourea (24 h)	57.25 ± 0.26*

*p < 0.001, compared to untreated gingival proteoglycan controls.

Analysis of gingival proteoglycans exposed to ROS for 24 h indicated that H_2_O_2_ treatment alone resulted in a limited loss of GAG content overall, with only ∼0.2% loss following 20 mM H_2_O_2_ (*p* > 0.05) and ∼13% loss following 60 mM H_2_O_2_ (*p* < 0.001) treatments over 24 h, compared to untreated controls (Figure 3(B)) and Table 1. The largest GAG loss percentage was evident in gingival proteoglycans exposed to an ·OH flux for 24 h, where virtually all GAG content was lost (∼83%, *p* < 0.001, Figure 3(B)) and Table 1. Again, the additional presence of thiourea partly alleviated ·OH-induced GAG loss (∼57%, *p* < 0.001), although GAG loss was still significant compared to untreated gingival proteoglycans (*p* < 0.001, Figure 3(B)) and Table 1.

### ROS effects on gingival proteoglycan core proteins by amino acid analysis

The amino acid compositions of gingival proteoglycan core proteins following exposure to ROS for 24 h, are presented in Table 2. Ovine gingival proteoglycans were demonstrated to contain all major amino acids, except cysteine and tryptophan. The core proteins of these proteoglycans were found to be acidic in nature, being rich in aspartate, glutamate, glycine, and leucine. Gingival proteoglycan exposure to increasing H_2_O_2_ concentrations over 24 h, led to elevations in the detectable levels of serine, glycine, and alanine residues, with no considerable changes in the detection of the other amino acids. However, exposure of the gingival proteoglycans to an ·OH flux for 24 h induced major decreases in glutamate, proline, isoleucine, and leucine levels, with corresponding increases in the detectable levels of serine, glycine, and alanine residues (Table 2). The amino acid, methionine, was also completely depleted. In contrast, the contents of the other amino acid residues detected remained relatively unchanged.

**Table 2.** Amino acid analysis of gingival proteoglycan core proteins exposed to ROS for 24 h, compared to untreated proteoglycan controls (*n = 1*).

Amino acid	Untreated	+ 20 mM H_2_O_2_	+ 60 mM H_2_O_2_	+ ·OH Flux
Aspartate	103	96	91	76
Threonine	64	65	60	58
Serine	73	76	95	289
Glutamate	129	123	117	49
Proline	54	60	39	ND
Glycine	96	99	111	212
Alanine	70	72	89	113
Cysteine	ND	ND	ND	ND
Valine	62	62	60	39
Methionine	2	4	6	ND
Isoleucine	60	61	61	22
Leucine	147	149	145	36
Tyrosine	21	19	20	16
Phenylalanine	35	35	32	16
Lysine	29	29	27	16
Histidine	20	15	15	42
Tryptophan	ND	ND	ND	ND
Arginine	35	35	32	16

Results expressed as residues per 1000. ND, not detected.

## Discussion

The present study has examined the modification and degradative effects of ROS, in particular H_2_O_2_ and ·OH species, on gingival proteoglycans associated with the ovine periodontium. Although gingival proteoglycan susceptibility to ROS-induced reductions in specific viscosity and molecular size has previously been reported [46], this is the first study to investigate the precise effects of contrasting ROS exposure on the core protein and GAG chain constituents of these proteoglycan molecular structures, to obtain a better understanding of their potential roles as an underlying mechanism of ECM destruction during active periods of gingivitis and periodontal diseases overall.

Biochemical characterization of the purified ovine gingival proteoglycans identified dermatan sulfate as the predominant GAG species, with smaller quantities of chondroitin sulfate and heparan sulfate also present. The proteoglycan core proteins were further demonstrated to be rich in aspartate, glutamate, glycine, and leucine residues. Similar GAG and core protein profiles have previously been reported for gingival connective tissues derived from other species, being associated with the principle presence of the SLRP, dermatan sulfate-substituted, decorin; and the large aggregating, chondroitin sulfate/dermatan sulfate proteoglycan, versican [29–38]. Other SLRPs are also recognized to be present in gingival connective tissues, including dermatan sulfate-substituted, biglycan; and keratan sulfate-containing, lumican, and fibromodulin [37,38]. However, these are present in much lower quantities compared to decorin and versican. Furthermore, the gingival epithelial tissues have been shown to predominantly consist of heparan sulfate-containing syndecans; in addition to the SLRPs, decorin, biglycan, fibromodulin, and lumican, in lesser quantities [30–32,35,37,39–41]. Therefore, despite the established presence of heterogeneous proteoglycan populations within gingival connective and epithelial tissues from various sources, it is likely that the structural modifications to proteoglycan core proteins and GAG chains presented in this study, primarily reflect changes in the decorin, versican, and syndecan proteoglycan species present within gingival tissues.

Detailed analysis of the respective core protein and GAG chain constituents of gingival proteoglycans following ROS treatment, determined that the gingival proteoglycans remained relatively intact following exposure to H_2_O_2_ over 24 h, as assessed by the hexuronic acid profiles of the constituent GAG chains and gel filtration chromatography. In contrast, the proteoglycans underwent more extensive decreases in molecular size following ·OH treatment, especially following 24 h exposure. However, upon analysis of the GAG contents of ROS-exposed gingival proteoglycans by cellulose acetate electrophoresis, it was evident that the GAG contents remained relatively consistent following H_2_O_2_ exposure alone over 24 h, with more extensive GAG chain loss resulting from ·OH depolymerization. This was again particularly apparent following 24 h ·OH flux exposure, where virtually all GAG content was lost. Overall, these findings suggested that the GAG chains were not the main sites of gingival proteoglycan susceptibility to degradation by H_2_O_2_ alone, although more extensive ·OH exposure induced significant GAG chain degradation and loss. As such, it may be presumed that the limited gingival proteoglycan degradation detected in the presence of H_2_O_2_ alone, was principally mediated via the modification and degradation of the proteoglycan core proteins. Such conclusions are supported by previous reports on proteoglycans derived from other sources, indicating the abilities of O_2_·^−^, H_2_O_2_ and other ROS, such as hypochlorous acid (HOCl), to promote amino acid modification and cleavage of the core proteins of the large aggregating, chondroitin sulfate/keratan sulfate cartilage proteoglycan, aggrecan, without extensive sulfated GAG chain depolymerization [47,48,54,55]. Similar conclusions of enhanced core protein susceptibility to H_2_O_2_ and ·OH treatments have also been demonstrated with the chondroitin sulfate-substituted SLRPs in alveolar bone, decorin and biglycan [45]; in addition to the heparin sulfate-containing, basement membrane proteoglycan, perlecan, following treatment with other oxidants, such as HOCl and peroxynitrite (ONOO^−^) [56,57]. However, depolymerization of GAG chains following exposure to ·OH species was accompanied by major reductions in proteoglycan molecular size [42–45,47,48,54–57]. Therefore, the findings presented herein suggest the gingival proteoglycans undergo similar mechanisms of ROS-induced modification and degradation as proteoglycans derived from other tissues, with proteoglycan core proteins being more prone to the effects of H_2_O_2_ alone, compared to sulfated GAG chains. However, both the core proteins and GAG chains are highly susceptible to degradation by ·OH species, especially following prolonged exposure.

Much research has been performed into elucidating the precise mechanisms and oxidative products induced by ·OH species on GAG chain structures [27]. Sulfated GAG chains are established to be less susceptible to ROS modification and degradation than non-sulfated GAGs, such as hyaluronan [42–46]. Furthermore, whilst the hexuronic acid regions of GAG chains are more vulnerable to ·OH attack than hexosamine residues, even the non-sulfated hexuronic acid regions of sulfated GAGs, such as heparin, have been reported to be more susceptible to modification than the N-acetylglucosamine and N-sulfated D-glucosamine regions [58–61]. Therefore, it is accepted that the presence of sulfated groups bestows a level of protection to GAG chains against ·OH-induced, depolymerization. The actual mechanism by which sulfate groups protect GAGs from ·OH degradation has been suggested that the negative charges of the sulfate groups may be responsible for this resistance, permitting the binding of iron and copper ions, thereby limiting ·OH production and GAG chain depolymerization [62,63]. Consequently, radical generation at non-sulfated sites within GAG chain structures is mainly responsible for GAG chain fragmentation.

The hypothesis that core protein modifications by ROS were particularly responsible for decreases in gingival proteoglycan molecular size, was supported by amino acid analysis. The extensive degradation of gingival proteoglycans following prolonged ·OH flux exposure had a prominent effect on the amino acid composition of the core proteins. The amino acid analysis identified major reductions in glutamate, proline, isoleucine, and leucine contents, with corresponding increases in the detectable levels of serine, glycine, and alanine residues. Such findings suggested that glutamate, proline, isoleucine, and leucine were the most susceptible to modification and degradation by an ·OH flux. Of particular interest was the loss of leucine residues, as SLRPs, decorin, biglycan, lumican, and fibromodulin, all contain leucine-rich repeat sequences which are implicated in the orchestration of specific functions, such as collagen fibrillogenesis [28,29].

Proteins are well-established to be highly susceptible to ROS-induced damage, mediated via amino acid modification which leads to increased cross-linking, aggregation, peptide bond cleavage, and fragmentation; which ultimately influences protein structure and functions [64,65]. Amino acid modification by ROS results in the formation of numerous oxidized by-products, although many aromatic (phenylalanine, tyrosine) and non-aromatic (cysteine, proline, histidine, lysine, arginine, serine) amino acids are recognized to be particularly susceptible. Furthermore, ROS modification of certain amino acids has been shown to produce other amino acids as end products, such as cysteine, histidine, tryptophan, serine, and threonine residue conversion to glycine and alanine; or proline and histidine residue conversion to glutamate and aspartate, respectively [12,45,64,65]. Although gingival proteoglycans did not exhibit any extensive loss of amino acids following H_2_O_2_ exposure, the elevated levels of serine, glycine, and alanine residues detected imply that a degree of amino acid modification had occurred with H_2_O_2_ treatment alone. Therefore, such conclusions reinforce the concept that highly susceptible amino acid residues within the core protein structures of gingival proteoglycans, such as glutamate, proline, isoleucine, and leucine, are the prominent regions of peptide bond cleavage and protein degradation following ROS exposure.

Intriguingly, however, there were certain proteoglycan core protein amino acids, such as methionine, histidine, and tyrosine, whose susceptibilities to ROS-induced modification are well-recognized, but which did not exhibit extensive residue loss upon ROS exposure. Methionine oxidation is reported to be much slower when exposed to H_2_O_2_ alone compared to other sulfur-containing amino acids, such as cysteine; with susceptibility dependent on specific ROS encountered [58,59]. In line with our previous work with alveolar bone proteoglycans [45], gingival core protein levels of methionine are also generally very low overall. Thus, the subtle increases in methionine residues observed between untreated and H_2_O_2_-treated samples may partly be a consequence of technique or data analysis sensitivity. However, as evident herein, previous studies have shown that methionine residue levels are maintained in the core proteins of alveolar bone proteoglycan exposed to H_2_O_2_ alone, although methionine residues are undetectable due to extensive modification following ·OH exposure, via H_2_O_2_/Fe^3+^ generation [45]. Similarly, the retention of histidine residue was also a feature of alveolar bone proteoglycan core proteins exposed to H_2_O_2_ and ·OH fluxes [45], despite histidine residues being highly susceptible to modification by these ROS [58,59]. However, although tyrosine residues significantly declined following alveolar bone core protein degradation by ·OH fluxes [45], similar reductions were not evident herein. Although we can only speculate at present as to the reasons for the limited loss of histidine and tyrosine residues to ROS modification and degradation in gingival proteoglycan core proteins, it is plausible that such residues are particularly localized in the proximity of the GAG attachment sites within the proteoglycan structures, which potentially bestow certain levels of protection from ROS modification to amino acids within these regions, due to the presence of the sulfated GAG chains [42–45].

As alluded to above, the present study has utilized an *in vitro* model system composed of supra-physiological (mM) oxidant concentrations, in order to study the mechanisms by which ROS modify and degrade gingival proteoglycans. Similar approaches have previously proven beneficial in elucidating the mechanisms by which ROS modify and degrade proteoglycans (at mg/mL concentrations) purified from various other tissues, including cartilage [47,48] and alveolar bone [45]. Thus, the H_2_O_2_ and Fe^3+^ concentrations implemented herein to induce proteoglycan modification/degradation can be regarded as being beyond those expected *in vivo* within the tissues and biofluids of the oral cavity which would limit the physiological significance of our findings, for instance, compared with the μM H_2_O_2_ concentrations detectable in saliva [66,67]. Nonetheless, the data presented does provide novel insights into the relative susceptibilities of the core protein and GAG chain constituents of gingival proteoglycans to ROS overall, identifying that these proteoglycan species exhibit a similar mechanism of modification and degradation to proteoglycans from other tissues, such as alveolar bone and cartilage [45,47,48].

Despite the present study indicating that gingival proteoglycan core proteins are predominantly affected by ROS, even minor changes induced by ROS within the GAG chain structures could further have potentially detrimental consequences on the architecture, integrity, and functionality of the gingival ECM. Indeed, there are ever-increasing numbers of core protein- and GAG chain-mediated functions attributed to prominent gingival proteoglycans, decorin, versican, and syndecans, including the modulation of ECM component interactions, coordinating collagen fibrillogenesis, growth factor sequestration; and in the regulation of inflammation, cell signaling, attachment, migration, and proliferation [28,29]. Thus, the ROS modification/cleavage of core proteins and the depolymerization of GAG chains may not be of importance to ECM destruction associated with gingivitis, but may also have further implications for the ECM during gingival tissue homeostasis and repair. Indeed, the inter-relationship between ROS and proteoglycan/ECM biology has been proposed to impact the development of various human pathologies, including chronic inflammatory diseases [68,69].

Numerous studies have demonstrated the detrimental effects of active periodontal disease on the gingival ECM. Gingival connective tissue-associated, decorin and biglycan, have both been demonstrated to be markedly depleted in chronically inflamed periodontal tissues [70], with the core proteins of gingival proteoglycans established to undergo extensive degradation during gingivitis, whereas the proteoglycan GAG chains remain relatively intact [71–74]. However, there are currently conflicting reports on the impact of gingivitis on the content of gingival epithelial proteoglycans, such as syndecan-1 [41,75,76]. The only GAG reported to be comprehensively depolymerized in inflamed gingival tissues is hyaluronan. Such clinical findings are significant, as they provide evidence for the degradation of proteoglycan core proteins during chronic gingivitis, whilst the constituent sulfated GAG chains remain largely unaffected. Furthermore, previous studies have also confirmed that sulfated GAGs, such as chondroitin 4-sulfate, dermatan sulfate, and heparan sulfate, are more resistant to ROS depolymerization, compared to the high molecular weight, non-sulfated GAG, hyaluronan [42–46,58–61]. Therefore, such clinical manifestations are consistent with the findings of the present study and potentially provide further evidence to implicate inflammatory-cell-derived ROS in the pathogenesis of active gingivitis and periodontal diseases overall, complementing the bacterial and host enzymic mechanisms of periodontal tissue destruction, already established [3,7,9–11].
